# A comparative study of the influence of the deposition technique (electrodeposition versus sputtering) on the properties of nanostructured Fe_70_Pd_30_ films

**DOI:** 10.1080/14686996.2020.1780097

**Published:** 2020-07-13

**Authors:** Matteo Cialone, Monica Fernandez-Barcia, Federica Celegato, Marco Coisson, Gabriele Barrera, Margitta Uhlemann, Annett Gebert, Jordi Sort, Eva Pellicer, Paola Rizzi, Paola Tiberto

**Affiliations:** a Chemistry Department and NIS, University of Torino, Torino, Italy; b Metrology of Innovative Materials and Life Science, INRiM, Torino, Italy; c Institute for Complex Materials, IFW Dresden, Dresden, Germany; d Department of Physics, Autonomous University of Barcelona, Cerdanyola Del Vallès, Spain; e Catalan Institution for Research and Advanced Studies, Barcelona, Spain

**Keywords:** FePd alloy, electrodeposition, sputtering, thin films, magnetic properties, mechanical properties, stripe domains, perpendicular magnetic anisotropy, 105 Low-Dimension (1D/2D) materials, 106 Metallic materials; 203 Magnetics / Spintronics / Superconductors, 301 Chemical syntheses / processing; 303 Mechanical / Physical processing, 306 Thin film / Coatings, 503 TEM, STEM, SEM, 504 X-ray / Neutron diffraction and scattering

## Abstract

Sputtering and electrodeposition are among the most widespread techniques for metallic thin film deposition. Since these techniques operate under different principles, the resulting films typically show different microstructures even when the chemical composition is kept fixed. In this work, films of Fe_70_Pd_30_ were produced in a thickness range between 30 and 600 nm, using both electrodeposition and sputtering. The electrodeposited films were deposited under potentiostatic regime from an ammonia sulfosalicylic acid-based aqueous solution. Meanwhile, the sputtered films were deposited from a composite target in radio frequency regime. Both approaches were proven to yield high quality and homogenous films. However, their crystallographic structure was different. Although all films were polycrystalline and Fe and Pd formed a solid solution with a body-centered cubic structure, a palladium hydride phase was additionally detected in the electrodeposited films. The occurrence of this phase induced internal stress in the films, thereby influencing their magnetic properties. In particular, the thickest electrodeposited Fe_70_Pd_30_ films showed out-of-plane magnetic anisotropy, whereas the magnetization easy axis lied in the film plane for all the sputtered films. The domain pattern of the electrodeposited films was investigated by magnetic force microscopy. Finally, nanoindentation studies highlighted the high quality of both the sputtered and electrodeposited films, the former exhibiting higher reduced Young’s modulus and Berkovich hardness values.

## Graphical Abstract

**Figure:**
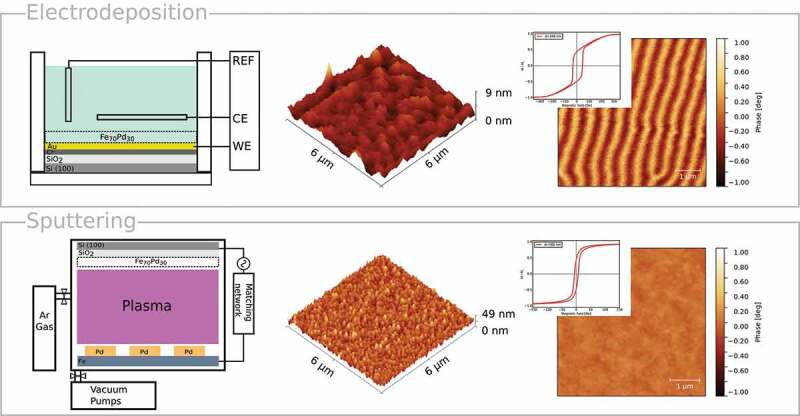

## 1. Introduction

Iron-palladium alloys arise great attention from the technological viewpoint as they show a unique and very interesting combination of mechanical and magnetic properties. As a result, they find uses in magnetic recording media [1–3], as microactuators and microsensors [4,5] or in spintronics [6]. In particular, the Fe_70_Pd_30_ (at.%) alloy shows the so-called ferromagnetic shape memory effect, related to the occurrence of a martensitic-to-austenitic phase transition [7,8]. This property is particularly appealing because, as compared to standard non-magnetic shape memory alloys, it allows for wireless magnetic manipulation. The possibility to induce the re-orientation of the twin boundaries in the martensite phase upon applying a magnetic field [9,10] makes it an excellent candidate for wirelessly actuated micro- and nanoelectromechanical systems (MEMS/NEMS) or strain sensors [11].

Current trends towards miniaturization of materials and devices require the use of appropriate deposition techniques. To date, Fe_70_Pd_30_ alloy films have been produced by both physical and (electro)chemical methods. Concerning physical methods, molecular beam epitaxy [12], sputtering [13,14] and pulsed laser deposition [15], are among the most common approaches. Their major drawback is the need of ultra-high vacuum and high temperature for deposition. In comparison, electrodeposition represents a fast and cost-effective alternative for the production of Fe_70_Pd_30_ thin films [16]. Moreover, unlike physical vapour deposition (PVD) methods, high aspect ratio structures are available by electrodeposition.

For a given material composition, comparative studies on the morphological and structural characteristics of films derived from physical and (electro)chemical deposition methods are rather scarce in literature [17–19]. Although the pros and cons of the different deposition methods are well known by the scientific community, specific considerations may apply to the particular system under investigation, and such features cannot be straightforwardly generalized. This is particularly true when it comes to electrodeposition. Although electrodeposition is commonly referred to as a cost-effective, faster technique which can coat larger substrate sizes compared to physical and chemical vapour deposition techniques, many film properties (roughness, crystal structure, type of defects, etc.) largely depend on the specific parameters applied to grow the material. On the other hand, thin metallic layers deposited on smooth substrates by co-sputtering (thickness generally in the submicron range) are characterised by an almost fully dense and uniform structure. Since microstructure and physical properties (e.g., magnetic and mechanical) are tightly correlated, alloy films produced from different deposition techniques might show very different properties.

The choice of the appropriate deposition technique is thus of utmost importance at the material design stage. Optimal selection requires a profound knowledge of the influence of the deposition techniques on the material characteristics in order to guarantee or be closer to the target properties. Although studies on the influence of the substrate type and the deposition conditions on the properties of Fe-Pd films, and of Fe_70_Pd_30_ alloy films, in particular, obtained by either electrodeposition [16,20,21] or sputtering [12,22] are available in the literature, a comparative study on the impact of the deposition technique (in particular, electrodeposition versus sputtering) is missing. In this work, this is addressed for Fe_70_Pd_30_ composition while trying to keep the rest of parameters involved in the processes (e.g. substrate type, film thickness) the same or as much comparable as possible.

## 2. Experimental

### 2.1. Sputtering

Sputtered films were produced via radio frequency (RF) sputtering from a composite target, where tiles of elemental palladium (purity 99.98%) are placed on top of an elemental target of iron (purity 99.99%). This configuration allows to change the exposed area of the two elements, which in turn allows to tune the stoichiometry of the deposited film. Fe_70_Pd_30_ (at.%) films were produced in the thickness range between 46 and 500 nm. The base pressure of the sputtering chamber was P*_base_* = (2.0 • 10^−7^) mbar, while during deposition the argon pressure was kept at P*_dep_* = (1.0 • 10^−2^) mbar. The substrate was a (100) oriented silicon single crystal (500 *μ*m thick) covered with 400 nm amorphous SiO_2_. Before film deposition, Si/SiO_2_ substrates were cleaned with acetone, isopropanol and finally de-ionized water in an ultrasonic bath.

### 2.2. Electrodeposition

Since conductive substrates are required for electrodeposition, a 100 nm thick layer of gold (Au) was deposited on top of the Si(100)/SiO_2_ substrate. In order to ensure good adhesion, a thin layer of chromium (2 nm thick) was deposited between the SiO_2_ and the Au layer. Prior to electrodeposition, the substrates were cleaned consecutively with acetone, isopropanol and de-ionized water in an ultrasonic bath. As a reference electrode, a saturated calomel electrode (SCE) was used (V*_SEC_* = 224 mV vs. standard hydrogen electrode (SHE) at T = 25^º^C), while a thin foil of platinum was used as a counter electrode. The working and counter electrodes were mounted into a cylindrical electrochemical cell in a horizontal arrangement, as previously reported by Iselt et al. [23]. Due to the particular setup of the electrochemical cell, a portion of the Au surface was used for electrical connection and remained uncoated after electrodeposition. Depositions were performed potentiostatically at room temperature and without stirring the electrolyte in a HEKA potentiostat galvanostat PG 310. The electrolyte composition, taken from a previous work of Konczak et al. [16], is 0.01 M Pd(NH_3_)_4_ Cl_2_, 0.06 M sulfosalicylic acid (SSA), 0.05 M Fe_2_ (SO_4_)_3_ • 7 H_2_O, 0.3 M (NH_4_)_2_ SO_4_ (pH = 5). Considering the deposition rates for electrodeposition and sputtering processes, 1.1 nm/s and 0.1 nm/s, respectively, deposition times were adjusted on-demand to yield deposits of comparable thicknesses.

### 2.3. Characterizations

Morphology and stoichiometry of the films were studied using atomic force microscope (AFM), scanning electron microscope (SEM, FEI Inspect F) and transmission electron microscope (TEM, Jeol JEM-3010). The latter was equipped with an energy dispersive X-ray spectrometer (EDS). The crystallographic structure of the films was investigated by grazing incidence X-ray diffraction (GIXRD) on a Panalytical X’Pert PRO MPD using the Cu Kα radiation, at a grazing angle of 0.4^º^. The magnetic properties were investigated using avibrating sample magnetometer (VSM) from Lakeshore, at room temperature, up to a maximum field of 20 kOe. Magnetic force microscopy (MFM) was used to image the magnetic domain patterns. A Bruker Multimode V Nanoscope 8 microscope equipped with a fully non-magnetic head and scanner, and a commercial Bruker MESP-HR10 cantilever coated with Co/Cr hard magnetic alloy were utilized. The mechanical properties of the films were measured by nanoindentation using a pyramidal-shaped Berkovich-type diamond tip [24]. Indentation experiments were performed in raster across the sample surface and the values for the reduced Young’s modulus, E*_r_*, and the Berkovich hardness, H*_B_*, were determined as the average from ≈ 300 indentations for each film using the method of Oliver and Pharr [25]. A complete list of the samples synthesized both via electrodeposition and via sputtering and analyzed in this work is reported in Table 1.

**Table 1.** Summary of the samples produced and their thicknesses

Electrodeposited films	Sputtered films
30 nm	46 nm
100 nm	100 nm
305 nm	300 nm
340 nm	–
600 nm	500 nm

## 3. Results and discussions

### 3.1. Morphology and structural properties

#### 3.1.1. Sputtered films

**Figure 1.**
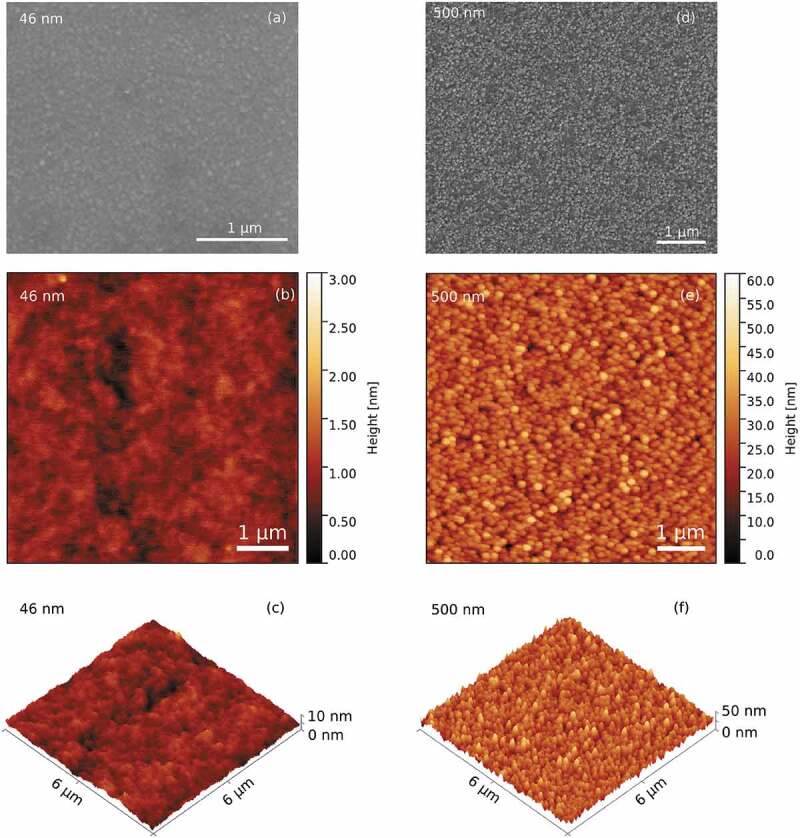
(a) SEM micrograph and (b) and (c) AFM images of the surface of the sputtered film with 46 nm thickness. (d) SEM micrograph and (e) and (f) AFM images of the surface of the sputtered film with 500 nm thickness

**Figure 2.**
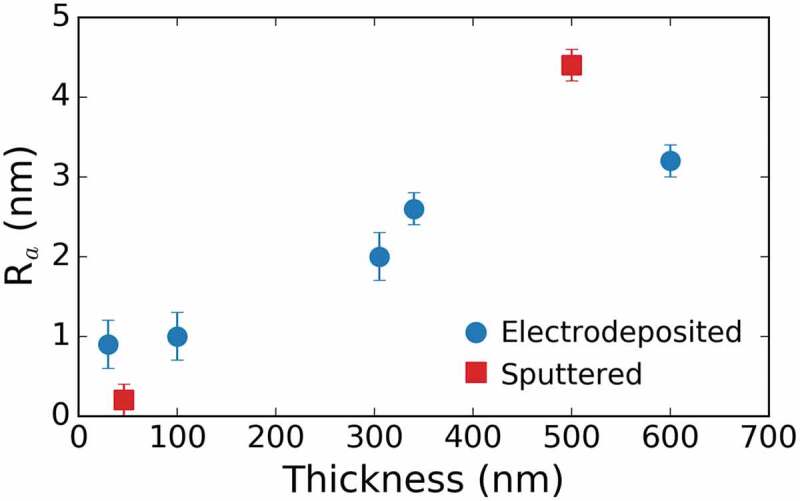
Evolution of the roughness for the sputtered and electrodeposited films as a function of the film’s thickness

Sputtered films with thicknesses ranging from 46 nm to 500 nm were produced. The on-top SEM and AFM images of the two extreme thicknesses, namely 46 nm and 500 nm, are shown in Figure 1. From the AFM images, the thickness-dependent surface morphology of the films is clear. Namely, the thinnest (46 nm) film has a roughness of R*_a_* = (0.2 ± 0.1) nm, while the thickest film (500 nm) has an R*_a_* = (4.4 ± 0.2) nm. The evolution of the roughness for both the sputtered and electrodeposited films as a function of the film’s thickness is summarized in the graph of Figure 2.

**Figure 3.**
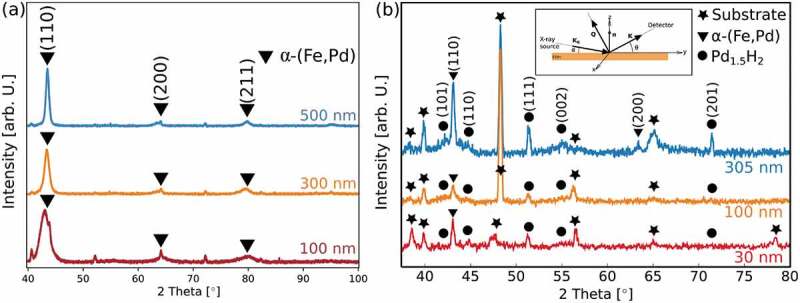
GIXRD plots labeled by film thickness from: (a) sputtered and (b) electrodeposited films. Intensity values are reported in linear scale. Unlabeled peaks in (a) belong to the substrate. Inset of panel (b) shows the geometry of the GIXRD set-up used for the acquisition of the GIXRD plots

The GIXRD curves of 100 nm, 300 nm and 500 nm thick sputtered films reported in Figure 3 (a) provide information on their crystal structure. Although the phase diagram predicts the occurrence of two phases, namely α-Fe + FePd, for the Fe_70_Pd_30_ composition [26], the GIXRD curves only show a set of reflections shifted to lower angles with respect to pure α-Fe. This might be an indication that the sputtered films are made of a single supersaturated solid solution of α-(Fe,Pd) [27]. Moreover, the (110) peak in the curves of Figure 3 (a) is broader (larger full width at half maximum) which can be related to a non-uniform distribution of Pd into the supersaturated solid solution [27]. Considering the relative intensity of the diffraction peaks, no preferential orientation is observed. Note that unlabeled peaks belong to the substrate. In the inset of Figure 3 (b) is sketched the geometry of the GIXRD configuration used to acquire the diffractions plots for the different films. This particular geometry, where the incoming X-ray beam is kept at a fixed small incidence angle (α) while the detector is rotating around the sample (with an angle *θ*, with *θ* ≠ α), implies that the scattering vector **Q** has a different direction for each different position (e.g. for each different θ) of the detector and it is not always perpendicular to the sample surface. Hence, the detected vector **K** has components along x, y and z-direction. In this case, the Scherrer’s equation can be used to estimate the overall dimensions of the crystalline grains. In particular, the Scherrer’s formula was applied on the (110) peak’s width to have a rough estimation of the crystal size, < D >, of the α-(Fe,Pd) phase (Table 2). < D > increased from 5 nm to 15 nm with film thickness, proving the nanocrystalline character of the sputtered films. Worth it to notice that the value of the < D >, obtained with the Scherrer’s formula, are generally underestimated. Indeed, in thin films the estimated values of crystallite size should be taken with caution.

**Table 2.** Values for the angular position (2 %\θ%), full width at half maximum (FWHM) and average crystal size < D > for the sputtered films, the latter calculated by applying the Scherrer’s formula on (110) peak width of the α -(Fe,Pd) phase

Thickness (nm)	2 θ (º)	FWHM (º)	< D > (nm)
100	43.08 ± 0.02	1.67 ± 0.02	5 ± 1
300	43.40 ± 0.02	0.87 ± 0.02	10 ± 2
500	43.51 ± 0.01	0.55 ± 0.01	15 ± 1

#### 3.1.2. Electrodeposited films

**Figure 4.**
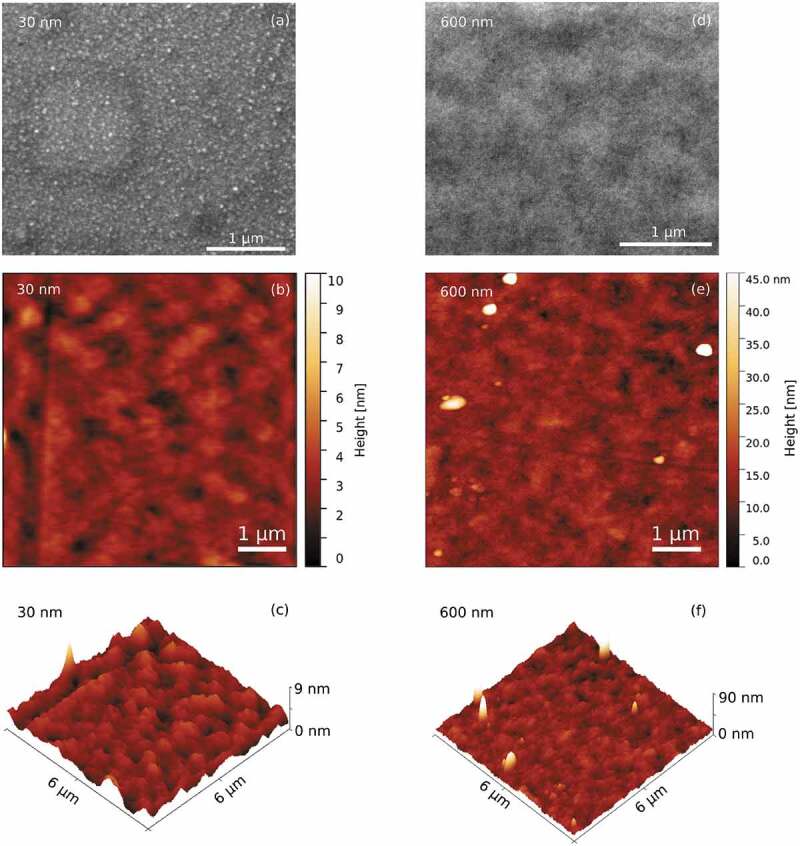
(a) SEM micrograph and (b) and (c) AFM images of the surface of the electrodeposited film with 30 nm thickness. (d) SEM micrograph and (e) and (f) AFM images of the surface of the electrodeposited film with 600 nm thickness

For comparison purposes, electrodeposited films with thicknesses ranging from 30 nm to 600 nm were produced. Importantly, complete coverage of the substrate was attained even in the 30 nm thick coating. Notice that complete coverage of the substrate is often not possible by electrodeposition for nanoscale thicknesses depending on the growth mode exhibited by the metal or alloy to be plated. As for the sputtered films, the on-top SEM and AFM images of the two extreme thicknesses, 30 and 600 nm, are depicted in Figure 4. The roughness also increases with film thickness. The thinnest film (30 nm) has a roughness of R*_a_* = (0.9 ± 0.2) nm, whereas R*_a_ *= (3.2 ± 0.2) for the thickest film (600 nm). These values are relatively small for electrodeposited films, which are often rougher than films produced by physical means. Interestingly, no cracks or inhomogeneities are apparent from the images. Note that voids are often encountered in metallic electrodeposits due to hydrogen co-evolution. Likewise, cracks might develop due to layer dehydration in the SEM vacuum chamber. None of these were observed in our electrodeposited films, confirming that, under optimized conditions, electrodeposited films can compete with those produced by PVD techniques.

**Figure 5.**
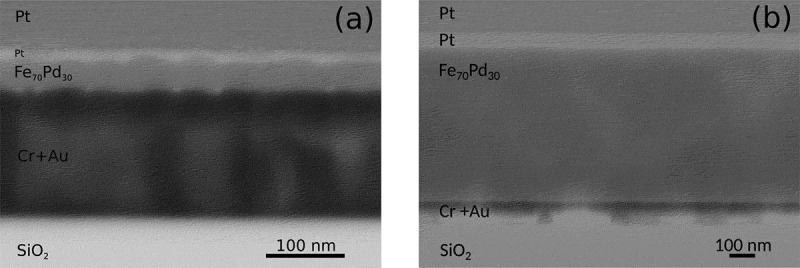
TEM images of the cross-section of the (a) 30 nm and (b) 600 nm thick electrodeposited films

In order to investigate the cross-section of the electrodeposited films, lamellas of the 30 nm and 600 nm thick films were prepared for TEM analyses and the results are shown in Figure 5 (a) and (b), respectively. The lamellas were prepared via the ion beam milling technique. Firstly, platinum is deposited on the surface of the film as a protective layer over the region of interest. Then, the region is deep-trenched all around, separated from the substrate, and via a nanomanipulator probe transferred and bonded to the TEM sample holder. Finally, the lamella is thinned down to a thickness of a few nanometers, using a low-intensity ionic current beam impinging on the lamella with a grazing angle [28]. From the cross-sections of Figure 5 (a) and (b) the presence of gross voids and cracks can be excluded. Nevertheless, given the magnification used, the presence of cracks and voids in the sub-nanometer range cannot be excluded. Furthermore, the composition of the films was determined along their cross-sections using spot-EDS analyses in the TEM. A homogeneous composition matching a Fe_70_Pd_30_ stoichiometry was observed for both thicknesses. Note that a platinum layer was deposited on top of the Fe_70_Pd_30_ films to protect the surface from oxidation during lamella fabrication by ion beam milling [28].

The GIXRD curves of electrodeposited films with varying thicknesses (30 nm, 100 nm and 305 nm) are shown in Figure 3 (b). Unlike the 100 nm and 300 nm thick sputtered films, the plots show reflections originating from two different crystalline phases, namely, *α*-(Fe,Pd) and tetragonal Pd_1.5_H_2_ phases. Interestingly, the presence of the tetragonal Pd_1.5_H_2_ phase is independent of film thickness, as it is detected even for the thinnest coating. Its relative amount, however, positively scales with film thickness. The formation of palladium hydrides is attributed to the high affinity between palladium and hydrogen [29,30]. This affinity is underpinned by the absorption of hydrogen atoms in the palladium crystal lattice. It was claimed that H:Pd ratios above 0.57 causes severe cracking of Pd deposits during or after electrodeposition because of significant lattice distortions [31]. However, cracking of the Fe_70_Pd_30_ deposits is not observed here. The crystal size of the electrodeposited films, as calculated by applying the Scherrer’s formula on the (110) peak’s width of the α-(Fe,Pd) phase, is around 31–32 nm (Table 3), hence larger than their sputtered counterparts. Nevertheless, the films are also nanostructured. Note that microstrains also contribute to peak broadening and thus the Scherrer’s formula underestimates crystal size.

**Table 3.** Values for the angular position, full width at half maximum (FWHM) and average crystal size < D > for the electrodeposited films, the latter calculated by applying the Scherrer’s formula on the (110) peak width of the α -(Fe,Pd) phase

Thickness (nm)	2 θ (º)	FWHM (º)	< D > (nm)
30	42.69 ± 0.05	0.26 ± 0.07	32 ± 4
100	42.92 ± 0.03	0.87 ± 0.02	32 ± 4
305	43.10 ± 0.05	0.27 ± 0.06	31 ± 5

### 3.2. Magnetic properties

**Figure 6.**
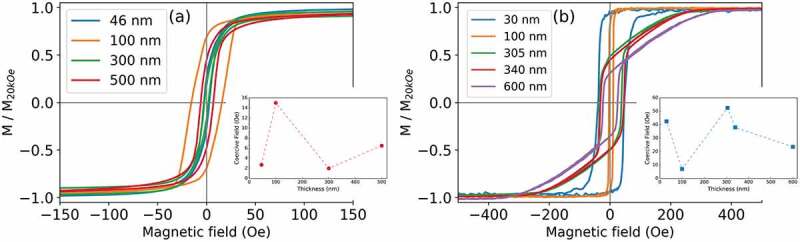
Hysteresis loops, measured at room temperature, for the in-plane orientation of the field corresponding to the (a) sputtered and (b) electrodeposited films with varying thickness. The insets show the evolution of the coercive field as a function of the film thickness for the sputtered and electrodeposited films

The magnetic hysteresis loops of Fe_70_Pd_30_ films produced by both electrodeposition and sputtering were measured at room temperature along the in-plane direction of the field. Different thicknesses were considered for both types of films and the results are shown in Figure 6 (a) and (b). The hysteresis loops of the sputtered films (Figure 6 (a)) are characterized by low values of coercivity, H*_C_*, and a sharp irreversible jump of the magnetization.

This behaviour is typical of soft magnetic materials. Moreover, the sputtered films are characterized by an in-plane anisotropy (i.e. the easy axis for the magnetization lies in the film plane) induced by the strong shape anisotropy due to the reduced film thicknesses. Different values of coercivity ranging from (3.0 ± 0.1) to (15.0 ± 0.3) Oe are observed, which do not vary linearly with film thickness. Dissimilar coercive field values can be understood considering that the dimensions and shapes of the crystalline grains change depending on film thickness [32,33]. Indeed, the magnetic properties of nanocrystalline films depend on the interplay between local magnetic anisotropy and exchange interactions, both of which are related to the size of the crystalline grains. For large grains, the magnetization tends to align along the magnetic easy axis direction of each grain. For large enough crystals, multiple domains can be generated [34]. Grain boundaries indeed represent an obstacle for the motion of domain walls, thereby hindering magnetization reversal and increasing coercivity [35]. In general, as the grain size is reduced, the coercive field first increases until it achieves a maximum and then it decreases [35,36]. The decrease in the value of coercivity for very small grain sizes is due to the enhanced exchange interactions between neighbouring crystalline grains [37,38]. Indeed, according to the random anisotropy model (originally proposed by Alben et al. [39]), for small grains, the exchange interaction forces the magnetic moments of different grains to align parallel to each other. As a consequence, the effective magnetic anisotropy is decreased (due to the random orientation of exchange coupled grains) and coercivity (which is proportional to the anisotropy) decreases as well [34]. The exchange interaction between neighbouring crystalline grains increases with decreasing crystal dimensions. The combination of these various effects determines the modulation of the H*_C_* values observed in the hysteresis loops of Figure 6 (a).

Regarding the hysteresis loops of the electrodeposited films, a markedly different behaviour is observed (Figure 6 (b)) as a function of film thickness. Both the 30 nm and the 100 nm thick films show a soft magnetic behaviour with in-plane anisotropy, similar to the sputtered films. These two films show different coercivity values, from (20.0 ± 0.4) to (40.0 ± 0.8) Oe which, as aforementioned, is ascribable to the different dimensions and hence, interactions between the crystalline grains. The shape of the hysteresis loops drastically changes for the thickest films (305 nm, 340 nm and 600 nm). These loops exhibit a sharp jump of the magnetization at small fields around the coercive field. When the value of the field is further increased, the magnetization increases linearly before reaching saturation, suggesting a rotation of the magnetization from the perpendicular direction towards the film plane. This particular shape of the loop is termed ‘transcritical loop’ [40,41], and indicates the existence of an out-of-plane anisotropy, hence, an out-of-plane component of the magnetization. The origin of this component of the magnetic anisotropy perpendicular to the film plane can be correlated with the presence of strains inside the electrodeposited film. Indeed, for the electrodeposited film, a small shift of the (110) peak of the α-(Fe,Pd) towards lower angles have been observed. This shift is an indication of a lattice distortion that can be ascribed to the presence of internal stresses in the electrodeposited films. Even if the available data do not allow elucidating the actual cause of the lattice distortion, it can be inferred that the presence of an additional phase in the electrodeposited films acts as a source of stresses in the deposit. Indeed, the PdH phase is embedded in the films, and because it has a lattice parameter (a_P_*_dH_* ≈ 2.99 Å) higher than the one of the α-(Fe,Pd) phase (a_α-(Fe,Pd)_≈ 2.87 Å), it acts as a source of internal stress.

Considering the magnetic peculiarities of the electrodeposited films, the configuration of their magnetic domain patterns was investigated by MFM at room temperature. The samples were previously saturated with an in-plane magnetic field and subsequently measured at magnetic remanence.

**Figure 7.**
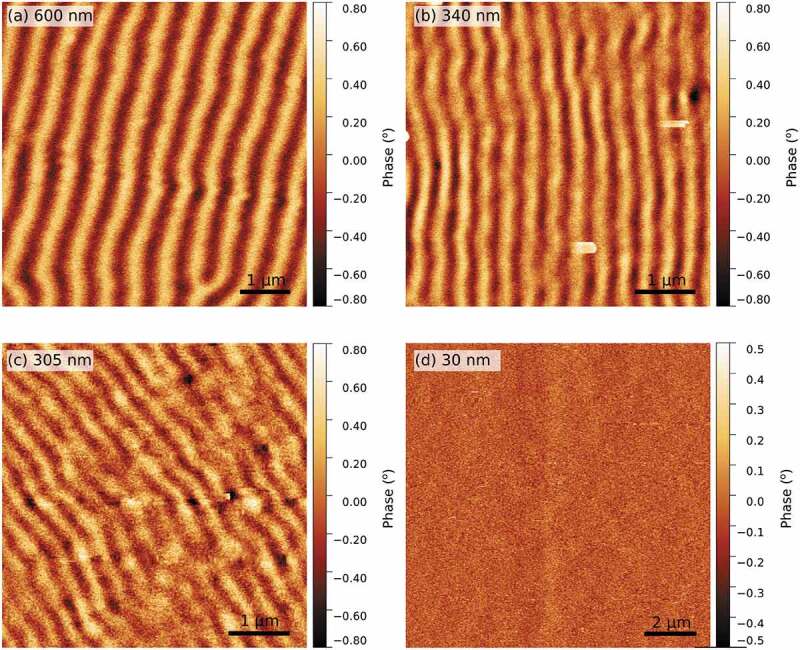
Room temperature MFM images, acquired at magnetic remanence, for the electrodeposited films with a thickness of (a) 600 nm, (b) 340 nm, (c) 305 nm and (d) 30 nm

The films displaying the transcritical hysteresis loop (305 nm, 340 nm and 600 nm thick films) show the typical stripe domain pattern, as can be seen in Figure 7 (a), (b) and (c). The dark and bright contrast is due to the magnetization canting up and down from the film plane [42]. Since MFM is only sensitive to the out-of-plane magnetization component, the occurrence of a non-uniform contrast in Figure 7 (a), (b) and (c) is a proof of the existence of an out-of-plane component of the magnetization. Conversely, Figure 7 (d) shows a uniform contrast, indicating that the magnetization is lying entirely in the film plane.

The domain pattern of the sputtered films was not investigated by MFM since the hysteresis loops of Figure 6 (a) show an in-plane anisotropy for all films. Hence, the MFM images would have a constant contrast, providing no information on the actual arrangement of the magnetic domains.

### 3.3. Mechanical properties

As aforementioned, the Fe_70_Pd_30_ alloy shows a combination of shape memory effect with ferromagnetic behaviour, which make thin films of this composition promising candidates for the design of wirelessly actuated MEMS/NEMS or strain sensors [11]. For this reason, it was deemed convenient to investigate and compare the nanomechanical properties of the sputtered and electrodeposited films. Indeed, as pointed by [1], the mechanical properties of Fe_70_Pd_30_ alloy films have been largely overlooked. This is particularly true when it comes to electrodeposited samples.

Similar to the magnetic properties, the mechanical response of the films can be correlated with the crystallographic phases and grain dimensions of the films [36]. Given the reduced thickness of the coatings, the mechanical properties were studied by nanoindentation using a standard Berkovich-type tip. Because surface roughness was very low, indentation could be performed from on top. The maximum indentation load was 0.3–0.4 mN, which was chosen to keep the penetration depth approximately 23–25 nm from the surface (i.e., below one-tenth of the total film thickness). Under these conditions, the mechanical properties of films thicker than 250 nm could be investigated. Specifically, sputtered and electrodeposited films having comparable thicknesses (300–305 nm and 500–600 nm) were subject to indentation.

**Figure 8.**
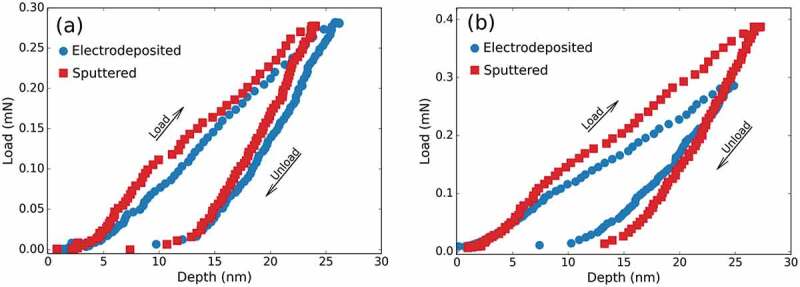
Load-displacement nanoindentation curves of the: (a) 305 nm thick electrodeposited (circles) and 300 nm thick sputtered (squares) films and (b) 600 nm thick electrodeposited (circles) and 500 nm thick sputtered (squares) films

The obtained results are summarized in Tables 4 and 5, while representative load–displacement curves are shown in Fig. 8 (a) and (b).

**Table 4.** Berkovich nanohardness (H*_B_*), reduced Young’s modulus (E*_r_*), H*_B_*/E*_r_*, H${\;}_{B}^{3}$/E${\;}_{r}^{2}$ and U*_e_*/U*_tot_* for sputtered Fe_70_Pd_30_ films of varying thickness (t)

t (nm)	E*_r_* (GPa)	H*_B_* (GPa)	H*_B_*/E*_r_*	H${\;}_{B}^{3}$/E${\;}_{r}^{2}$ (GPa)	U*_e_* (nJ)	U*_p_* (nJ)	U*_e_*/U*_tot_*
300	129 ± 3	6.4 ± 0.2	0.050 ± 0.002	0.015 ± 0.003	0.0015 ± 0.0003	0.0019 ± 0.0002	0.5 ± 0.1
500	162 ± 18	8 ± 1	0.05 ± 0.01	0.02 ± 0.01	0.0028 ± 0.0006	0.0026 ± 0.0002	0.4 ± 0.1

**Table 5.** Berkovich nanohardness (H*_B_*), reduced Young’s modulus (E*_r_*), H*_B_*/E*_r_*, H${\;}_{B}^{3}$/E${\;}_{r}^{2}$ and U*_e_*/U*_tot_* for electrodeposited Fe_70_Pd_30_ films of varying thickness (t)

t (nm)	E*_r_* (GPa)	H*_B_* (GPa)	H*_B_*/E*_r_*	H${\;}_{B}^{3}$/E${\;}_{r}^{2}$ (GPa)	U*_e_* (nJ)	U*_p_* (nJ)	U*_e_*/U*_tot_*
305	105 ± 2	5.9 ± 0.1	0.056 ± 0.002	0.018 ± 0.001	0.0020 ± 0.0004	0.0022 ± 0.0002	0.5 ± 0.2
340	112 ± 12	6.5 ± 0.9	0.05 ± 0.01	0.02 ± 0.01	0.0016 ± 0.0002	0.0024 ± 0.0001	0.6 ± 0.1
600	115 ± 12	6.8 ± 0.9	0.06 ± 0.01	0.02 ± 0.01	0.0014 ± 0.0008	0.0022 ± 0.0002	0.6 ± 0.4

Overall, the sputtered films Fe_70_Pd_30_ show larger E*_r_* values than their electrodeposited analogues (cf. Tables 4 and 5). Although the latter looked very compact (see Figure 5), a slightly higher density can be claimed for the sputtered films. Since stiffness generally scales up with density, this would explain the higher E*_r_* values of the physically deposited films. Similarly, H*_B_* values are also higher. The E*_r_* and H*_B_* values for the sputtered films are in agreement with the literature. For example, H*_B_* = 7.3 GPa and E = 140 GPa were reported for Fe_70_Pd_30_ films grown by PVD on Si/SiO_2_ substrate. Chiu et al. obtained H*_B_ *= 12.5 GPa and E = 152 GPa for Fe_50_Pd_50_ films with a crystal size of 12 nm grown by magnetron sputtering [1]. An important observation is that for both sputtered and electrodeposited films, H*_B_* and E*_r_* values increase with thickness. This seems contradictory especially for the sputtered films since the crystal size of the α-(Fe,Pd) phase increases with thickness (see Figure 3 and Table 2). According to the Hall–Petch relationship, hardness is expected to increase with a decrease of crystal size due to dislocation pile-up at grain boundaries. Therefore, there has to be another factor which overcomes the apparent increase of crystal size with thickness. The reason behind this trend might be related to the relatively high hardness of the fabricated Fe_70_Pd_30_ films. Under conditions for which the coating is stiffer than the substrate, the one-tenth rule might not necessarily apply and, therefore, an influence from the Si/SiO_2_ (400 nm) substrate for the lowest Fe_70_Pd_30_ sputtered film thicknesses cannot be excluded [43]. A similar reasoning can be made for the electrodeposited films, for which the substrate is Si/SiO_2_ (400 nm)/Cr (2 nm)/Au (100 nm); the layer in direct contact with the electrodeposited film (Au) is definitely softer than the here-fabricated Fe_70_Pd_30_ coatings. There could be another factor contributing to the thickness-dependence of H*_B_* in the electrodeposited coatings. Since the relative amount of palladium hydrides increases with deposit thickness (see section Electrodeposited films) and these compounds are known to induce strain, progressively higher H*_B_* values would be expected as the film builds up. Tables 4 and 5 also list several meaningful ratios, namely, H*_B_*/E*_r_*, H${\;}_{B}^{3}$/E${\;}_{r}^{2}$ and U${\;}_{e}$/U${\;}_{tot}$. The former can be regarded as an indirect assessment of the wear resistance of a coating [44]. Results indicate that this parameter is similar for both sputtered and electrodeposited films and it is about 0.05–0.06. Similar hardness to reduced elastic modulus ratios were found in nanostructured Co-Ni-Re-P alloys fabricated by electrodeposition with 18 at.% of rhenium as refractory metal [45]. The H${\;}_{B}^{3}$/E${\;}_{r}^{2}$ ratio, which allows to estimate the material’s ability to dissipate energy at plastic deformation during loading, is about 0.02 (and a bit less for the thinnest films). Finally, the ratio between U*_e_* and U*_tot_* (elastic recovery) was estimated from the areas enclosed between the unloading indentation segment and the displacement axis (U*_e_*) and between the loading segment and the displacement axis (U*_tot_*). This ratio is of particular interest in impact loading applications since it indicates how much energy is released from the material after being loaded. Results indicate that the thickest electrodeposited films (340 nm and 600 nm) are able to dissipate more energy than their sputtered counterparts (500 nm). Indeed, U*_e_*/U*_tot_* and H*_B_*/E*_r_* values are exactly the same in some cases, as it happens in elastic perfectly plastic materials. Overall, the mechanical properties indicate that the electrodeposited Fe_70_Pd_30_ films are mechanically as good as or even superior to their sputtered analogues.

## 4. Conclusions

In this work, the magnetic and mechanical properties of nanostructured Fe_70_Pd_30_ films produced via potentiostatic electrodeposition and radio frequency sputtering were investigated and correlated with their microstructure. Both approaches were proven to produce homogeneous films, with roughness below 5 nm. The sputtered films, independently from their thickness, were single phase with a body-centred cubic structure belonging to a supersaturated solid solution of α-(Fe,Pd). The electrodeposited films show, alongside with the α-(Fe,Pd) phase, the presence of palladium hydrides. The Pd_1.5_H_2_ phase causes internal stresses in the electrodeposited film which, in turn, influences the magnetic properties of the material. In particular, the stresses induce out-of-plane magnetic anisotropy which results in a transcritical hysteresis loop and a striped magnetic domain pattern in films thicker than 100 nm. The presence of an out-of-plane anisotropy is relevant for a number of applications such as magnetic storage devices, sensors or spintronics. Regarding the mechanical behaviour, the sputtered films show high reduced Young’s modulus and Berkovich hardness values, which can be attributed to their compactness. Yet, the electrodeposited films looked rather dense by TEM and thereby a pronounced decrease of these parameters was not observed. The reduced Young’s modulus and Berkovich hardness were thickness dependent, indicating an influence from the underlying substrate for the lowest film thickness tested (300 nm). Interestingly, rather large H*_B_*/E*_r_* and H${\;}_{B}^{3}$/E${\;}_{r}^{2}$ ratios were obtained for both sets of samples, suggesting good wear resistance and the ability to dissipate energy during plastic deformation. Fundamental understanding of the influence of the deposition technique and film thickness on the physical properties of Fe_70_Pd_30_ films will help choose the right synthetic conditions in view of the end application.
